# Therapeutic Targeting of IRFs: Pathway-Dependence or Structure-Based?

**DOI:** 10.3389/fimmu.2018.02622

**Published:** 2018-11-20

**Authors:** Cherrie D. Thompson, Bharati Matta, Betsy J. Barnes

**Affiliations:** Center for Autoimmune Musculoskeletal and Hematopoietic Diseases, Feinstein Institute for Medical Research, Manhasset, NY, United States

**Keywords:** IRF5, inhibition, negative regulation, positive regulation, autoimmunity

## Abstract

The interferon regulatory factors (IRFs) are a family of master transcription factors that regulate pathogen-induced innate and acquired immune responses. Aberration(s) in IRF signaling pathways due to infection, genetic predisposition and/or mutation, which can lead to increased expression of type I interferon (IFN) genes, IFN-stimulated genes (ISGs), and other pro-inflammatory cytokines/chemokines, has been linked to the development of numerous diseases, including (but not limited to) autoimmune and cancer. What is currently lacking in the field is an understanding of how best to therapeutically target these transcription factors. Many IRFs are regulated by post-translational modifications downstream of pattern recognition receptors (PRRs) and some of these modifications lead to activation or inhibition. We and others have been able to utilize structural features of the IRFs in order to generate dominant negative mutants that inhibit function. Here, we will review potential therapeutic strategies for targeting all IRFs by using IRF5 as a candidate targeting molecule.

## Introduction

Interferon Regulatory Factors (IRFs) are a family of transcription factors that signal downstream of multiple pathways, including Toll-like receptor (TLR), retinoic acid-inducible gene I (RIG-I), melanoma differentiation associated gene 5 (MDA5), and B cell receptor (BCR) signaling pathways to regulate gene expression involved in both innate and adaptive immunity (1–3). IRFs are also known to play central roles in cell differentiation and development, cell proliferation, apoptosis, DNA damage response and tumor suppression (2–9). There are currently 9 mammalian IRFs-IRF1, IRF2, IRF3, IRF4/PIP/ICSAT, IRF5, IRF6, IRF7, IRF8/ICSBP, and IRF9/p48/ISGF3γ (3). This family of transcription factors is generally localized to the cytoplasm of an unstimulated cell, in which they exist in their inactive monomeric form. Induction of the different signaling cascades leads to the recruitment of adaptor molecules that in turn regulate a cascade of signals to promote IRF activation and nuclear translocation. This process ultimately leads to the downstream production of cytokines, chemokines and other transcription factors that regulate innate and adaptive immune responses (10, 11).

**Figure 1:**
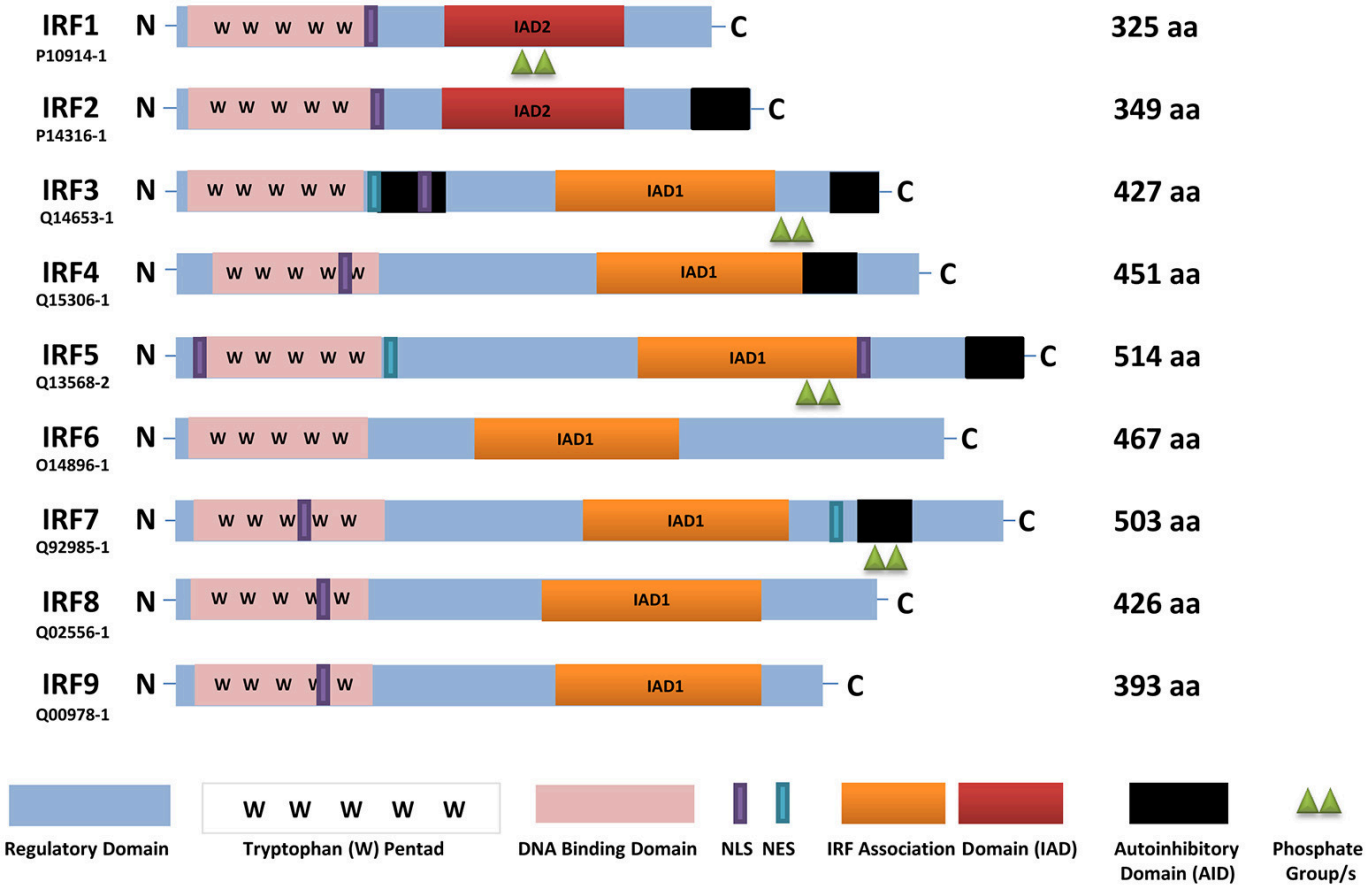
A schematic representation of full-length human IRFs showing different functional domains. All IRFs harbor a DNA binding domain that contains a conserved tryptophan pentad (pink) in the N-terminus. They also contain an IRF activation domain termed either IAD1 (orange) or IAD2 (red). Other domains present are a nuclear localization signal (NLS, purple), nuclear export signal (NES, blue-green), an autoinhibitory domain (black), and a regulatory domain (blue). In this scheme, IRF activation (green triangles) is denoted as phosphorylation. The length of each IRF is indicated by the number of amino acids (aa), as found in Uniprot, with each identifier listed. IRF, interferon regulatory factor; C, carboxy terminus; N, amino terminus.

A key event prior to IRF activation and nuclear translocation is post-translational modification that leads to conformational changes allowing for protein-protein interactions. In the case of IRFs that contain a carboxyl (C)-terminal autoinhibitory domain (AID) (Figure 1), post-translational modification leads to disruption of intramolecular association of the AID with the amino (N)-terminal DNA binding domain (DBD) and IRF association domain (IAD) (12–14). Ultimately, these conformational changes enable the IRFs to homo- or hetero-dimerize with each other or another molecule, thus allowing them to translocate to the nucleus and bind to DNA (with other co-factors), resulting in the regulation of gene transcription (15, 16). As in most critical signaling pathways that elicit an immune response, once the response has been elicited and immune cells respond, an intrinsic negative regulatory pathway is expected to be initiated to shut down the originating signal. If activation persists, inflammatory molecules will begin to damage tissues, and/or trigger the development of autoimmunity.

Indeed, hyper-activation of IRFs (most notably IRF1, IRF3, IRF5, IRF7, and IRF9) has been implicated in disease pathogenesis as it leads to unrestricted production of IFNs, which is linked to the development of numerous inflammatory and autoimmune diseases (17, 18). Further, polymorphisms in *IRF* genes show either protection from or increased susceptibility to the development of such diseases (19–23). Thus, the development of small molecules that directly bind to and inhibit IRF function(s) would be extremely valuable to patients with a variety of inflammatory and autoimmune diseases. To date, there are no therapeutic inhibitors of the IRFs. In general, transcription factors are thought to be notoriously difficult to target (24). This certainly holds true for IRFs as we still do not fully understand the physiologic mechanisms that control IRF activation and inhibition in a cell. For many IRF family members, the mechanism of activation depends on the cell type and initiating signaling pathway. Last, crystal structures of full-length IRFs have been difficult to resolve, which when done, will lend valuable insight into the rational targeting of specific structural features inherent to each family member (13, 14). Thus, indirect strategies for inhibiting IRF function(s) have been focused on by targeting molecules that regulate their activities, such as kinases that phosphorylate the IRFs, rather than directly targeting their structure.

Hence, in this review, we will discuss the critical events involved in IRF activation, including mechanisms of post-translational modification, classical IRF signaling pathways, and negative regulatory pathways as methods to indirectly target IRF activation and function. In addition, we will discuss new insights into the direct targeting of IRFs through focused studies on the IRF5 family member. Ultimately, understanding the mechanisms of IRF-mediated inflammatory responses will aid in the identification of new strategies to therapeutically target these critical players.

## Implications for IRFs in disease pathogenesis–why target the IRFs?

The role of IRFs and their importance in regulating immunity have been increasingly conspicuous in the last decade. Dysregulation of IRFs can lead to either suppression or hyper-activation, both of which may contribute to disease development. Hence, identifying methods to target the modulation of these transcription factors will provide new avenues of treatment for patients suffering from IRF-mediated diseases. In this section, we will briefly discuss IRF family members and their role(s) in disease pathogenesis.

IRF1 was the first family member to be identified and found to regulate type I IFN gene expression. Recent data from genome wide association studies (GWAS) identified IRF1 as a risk factor for inflammatory bowel disease (25, 26). In mice, IRF1 was shown to promote the severity and incidence of autoimmune diseases like collagen-induced arthritis (CIA) and experimental allergic encephalomyelitis. The incidence and severity of CIA and EAE were significantly reduced mice lacking *Irf1* (27).

Conversely, IRF2 is a negative regulator of IFN-mediated gene expression. IRF2 suppresses the activity of IRF1 by competing for binding sites (28). An increase in the IRF1/IRF2 ratio has been considered an important event needed for the transcriptional activation of IFNα genes required for the development of cellular responses to viruses (29). Limited and not very well-replicated studies have reported an association of *IRF2* polymorphism with susceptibility to the autoimmune disease systemic lupus erythematosus (SLE). The SLE risk haplotype was suggested to be associated with activation of IRF2 (17, 30, 31).

Similarly, *IRF3* polymorphisms were found to be associated with SLE but controversy still exists regarding their role in susceptibility and pathogenesis (17, 23, 32). Studies in a Mexican mestizo cohort found that the rs2304206 gene variant associated with increased IRF3 expression in plasmacytoid dendritic cells (pDCs), with elevated type I IFN expression and dsDNA autoantibodies (32). In a murine model of EAE, *Irf3*^−/−^ mice showed reduced disease severity due to attenuated Th1 and Th17 type responses (33). Further, IRF3 over-activation was found to contribute to autoinflammatory conditions, such as Aicardi-Goutières syndrome (34–36) and STING-associated vasculopathy of infancy (SAVI) (34, 36). Last, over-active IRF3 in macrophages and enhanced production of type I IFN resulted in fatal inflammatory response to myocardial infarction while *Irf3*^−/−^ mice were protected from myocardial infarction (36).

In contrast, dysregulated IRF4 has been implicate in multiple myeloma where its expression was found to correlate with malignancy-specific gene expression (37). *IRF4* polymorphisms were also found to contribute to elevated IRF4 expression in cells from multiple myeloma patients (38, 39). Polymorphisms in the *IRF4* gene have also been detected in adult T cell leukemia (40). Under the condition of chronic infection, IRF4 induces the exhaustion of CD8^+^ T cells and hinders the development of memory T cells (41). More recent findings suggest that *IRF4* polymorphisms are associated with high risk of rheumatoid arthritis (RA) (17, 42, 43) and systemic sclerosis (17, 43).

Mutations in *IRF6* have been shown to contribute to the development of Van der Woude syndrome (VWS) and popliteal pterygium syndrome (PPS). VWS is an autosomal dominant form of cleft lip and PPS is a disorder with a similar orofacial phenotype that includes skin and genital anomalies. Further, increased IRF6 mRNA was found along the medial edge of the fusing palate, tooth buds, hair follicles, genitalia and skin in samples with *IRF6* mutations (44, 45).

*IRF7* polymorphisms, like IRF5, are associated with increased risk of SLE (46–49). IRF7 has also been implicated in the pathogenesis of type 1 diabetes through the upregulation of inflammatory gene networks (50). Most relevant to the current review is the finding that reduction/inhibition of mucosal IRF7 expression with liposomal *Irf7* siRNA resulted in protection of mice from bacterial infection and renal tissue damage (51). Last, IRF7 expression was recently found to be elevated in PBMC from patients with systemic sclerosis, as compared to healthy donors, due to promoter hypomethylation (52).

IRF8 was recently found to play an important role in the differentiation of IL9-producing T helper cells (Th9). Th9 cells are a subset of CD4^+^ T cells with pro-inflammatory function (53). In the NZB/W F1 model of spontaneous murine lupus, mice lacking *Irf8* failed to produce anti-nuclear, -chromatin and -erythrocyte autoantibodies and had reduced kidney disease (54). Dual and opposing functions for IRF8 were found in Autoimmune Uveitis. Deletion of IRF8 in T cells exacerbated the disease, while loss of IRF8 in retinal cells had a protective effect (55). Additionally, a meta- analysis detected association of *IRF8* genetic variants with susceptibility of Multiple Sclerosis (MS) (56). Last, IRF8-expressing antigen presenting cells in EAE led to disease development by facilitating the onset and expansion of T effector cells and promoting microglial-based neuro-inflammation. Thus, mice lacking *Irf8* are protected from EAE (57).

Although limited reports implicate a direct role for IRF9 in disease pathogenesis that support its therapeutic targeting, IRF9 is well-known to regulate IFN signaling through formation of the ISGF3 complex (58). A recent report by Nan and colleagues, however, found that IRF9 contributes to STAT3 activation by upregulating IL6 expression in cancer cells. IL6 is necessary for some cancer cells to grow and thus inhibition of this pathway could be therapeutic (59).

We have saved IRF5 to discuss last as it has become the most widely implicated IRF in disease pathogenesis. In the last 10 years, numerous studies have reported the association of *IRF5* polymorphisms with autoimmune disease susceptibility. Diseases include, but are not limited to–RA, systemic sclerosis, MS, inflammatory bowel disease and SLE (17, 60–62). In the case of SLE, GWAS across multiple ancestral backgrounds have confirmed that *IRF5* polymorphisms associate with SLE risk [(60, 63–66)]. In SLE patient blood, IRF5 expression and activation were found to be significantly elevated (67–71). Prior to these findings, IRF5 was identified as a critical mediator of MyD88-dependent TLR signaling, leading to the expression/production of multiple pro-inflammatory cytokines including type I IFNs, IL6, TNFα, IL12, IL23, and others implicated in autoimmune disease pathogenesis (62, 72–76).

IRF5 has also been shown to play critical roles during viral infection. IRF5 was recently found to promote the death of protective CD4^+^ T cells during chronic visceral leishmaniasis resulting in the establishment of chronic infection (77). Expression levels of IRF5 and its related downstream inflammatory cytokines were also found to be associated with severity, prognosis, and the causative pathogen of community acquired pneumonia in patients (10). Last, genetic variants of *IRF5* have been associated with chronic hepatitis B infection (78).

In addition to its role(s) in autoimmune and viral disease pathogenesis, the past 5–10 years has brought about a plethora of new data implicating IRF5 in multiple other diseases, including cancer, obesity, neuropathic pain, cardiovascular, and metabolic dysfunction (79–82). For the purpose of this review, we will not be discussing the role of IRF5 in cancer as it tends to act as a tumor suppressor and thus its expression/activation are downregulated (83–87). We instead focus on diseases where IRF5 expression/activation are upregulated. For example, in two distinct models of murine atherosclerosis, murine *Irf5* was recently found to contribute to the formation of atherosclerotic lesions by impairing efferocytosis (88). This effect was due to IRF5's role in promoting the maintenance of pro-inflammatory CD11c^+^ macrophages within lesions leading to the expansion of the necrotic core. IRF5 also plays a role in liver fibrosis caused by hepatitis C virus or in non-alcoholic fatty liver disease (89). IRF5 expression was significantly higher in liver macrophages from human subjects with liver fibrosis than healthy controls and its expression positively correlated with clinical markers of liver damage. Of note, mice lacking *Irf5* in their myeloid compartment were protected from hepatic fibrosis (89). In a coronary ligation model, high levels of IRF5 expression were detected during the early inflammatory stage (day 4) of wound healing. This phase was then followed by a decrease in IRF5 expression in infarct macrophages skewing them toward an M2 phenotype that is involved in the resolution of inflammation (day 8). Accelerated cutaneous and infarct healing, and attenuated development of post-myocardial infarct heart failure were observed during the second phase of decreased IRF5 expression (81, 90).

IRF5 dysfunction was also recently implicated in neuropathic pain, which plays an important role in the pathogenesis of tactile allodynia induced by nerve injury. IRF5 expression on M1 microglia is upregulated by spinal nerve injury, which in turn induces the expression of ATP receptors to activate microglia and signal neuropathic pain in the spinal cord (91). In spinal cord injury (SCI) there is an acute, long-lasting inflammatory response and macrophages play an important role in persistent inflammation contributing to the pathogenesis of SCI. The first phase after SCI is acute and is characterized by M2 macrophage infiltration that is then followed by a long-lasting phase of M1 macrophages, which slows healing and compromises organ function. IRF5 was shown to play a critical role in this process by up-regulating genes associated with the M1 macrophage phenotype (92).

In the antigen-induced model of arthritis, a population of *Irf5*-positive pro-inflammatory macrophages was found to significantly increase in inflamed knees, suggesting that IRF5 can be used as a marker of inflammatory macrophages in a disease setting (93). Another report from the same group studied the role of IRF5 in a model of acute inflammation and lung injury. Neutrophil influx is known to play a major role in both diseases. Mice lacking *Irf5* had a significant reduction in the number of neutrophils accumulating at the site of infection, and acute lung injury was markedly reduced in *Irf5*-deficient mice (93).

Another important role for IRF5 was identified in patients carrying *IRF5* polymorphism rs3757385 that associates with acute rejection and is considered a risk factor for transplant rejection (94). *IRF5* polymorphisms were also recently identified that associate with asthma and its severity. Interestingly, *IRF5* risk alleles that associate with asthma were found to be almost completely opposite to those for autoimmune disorders, supporting potentially distinct roles for IRF5 in the pathogenesis of asthma and autoimmune disorders (95). Additional work in both human and mouse models of asthma and allergic airway inflammation suggests an important role for Irf5 in driving disease severity (96, 97).

A final example of IRF5 dysregulation in disease comes from the field of hematologic malignancies. Distinct from the multitude of solid cancers and hematologic malignancies that have been shown to have lost IRF5 expression (79, 83, 98), a tumor-promoting role for IRF5 was identified in classical Hodgkin Lymphoma (HL) where IRF5 expression was found to be elevated and over-activated in HL B cells (84, 99).

Given the multitude of studies implicating IRF5 dysregulation in a vast number of diseases, we use this IRF family member as a candidate therapeutic target for drug discovery. Below, we focus on the details of IRF5 structure-function, signaling, post-translational modification and negative regulation that may be used as molecular targets for therapeutic inhibition. Since there is significant homology between IRF family members (Figure 1), combined with distinct and overlapping functional roles in the immune system, we anticipate that strategies developed to inhibit IRF5 may be utilized to modulate the function/activity of other IRF family members.

## Understanding the molecular structure of IRF5

IRF family members regulate IFNs and IFN-inducible genes supporting their critical role(s) in the innate immune response against pathogens. All IRFs have a homology of over 115 amino acids in their N-terminal region that harbors the DBD (Figure 1). The DBD contains a highly conserved tryptophan (W) repeat forming a helix-turn-helix motif that recognizes DNA sequences referred to as IFN-stimulated response elements (ISRE) (A/GNGAAANNGAAACT) or IRF elements (IRF-E) (5, 9, 17). The C-terminal region, on the other hand, exhibits diversity in all IRFs, which supports their distinct function(s), and could be potentially used for therapeutic inhibition that would provide specificity to each family member. As summarized in Figure 1, the IRFs contain a regulatory domain, nuclear localization signal (NLS), nuclear export signal (NES), IRF-association domains (IAD), and some family members (IRF3, IRF5, IRF7) contain an autoinhibitory domain (AID) (48, 100). Each of these regions defines or elicits cell type-specific functions, activation via distinct signaling pathways, and interaction with other proteins.

**Figure 2:**
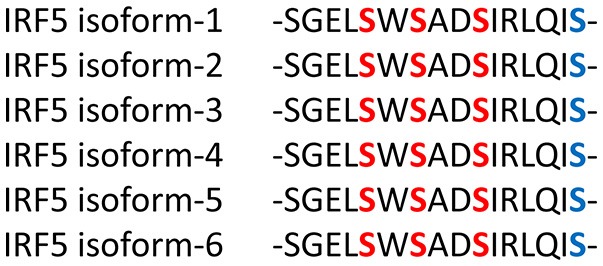
The Serine Rich Region (SRR) is conserved in all IRF5 isoforms. The C-terminus contains conserved serine (**S**) residues that are targeted for phosphorylation by kinases, such as IKKβ (blue-bolded serine). Red-bolded serines are those originally identified as critical for IRF5 activation (101, 13). Phosphorylation leads to structural changes, including removal of the AID, liberation of the IAD and exposure of the C-terminus for further modification(s) and/or protein interaction. Although IRF5 isoforms range in size, most contain the SRR independent of its numerical amino acid location.

The AID suppresses IRF transcriptional activity. There are two identified AIDs in IRF3 located in the N- and C-terminal regions compared to one AID found in IRF5 and IRF7 (14, 101, 102). The IRF3 crystal structure in its latent (unstimulated or autoinhibited monomer) form revealed the hydrophobic surface and a region essential for CBP/p300 binding that is masked by the AID (14). The presence of two AIDs provides a unique activation conformation upon phosphorylation with the IAD and AID forming a hydrophobic core and realignment of the DBD. The pseudo-phosphorylated IRF5 crystal structure, on the other hand, revealed the AID and key phosphorylation sites (Figure 2) as being highly extended allowing for dimerization and/or interaction with CBP/p300 in the hydrophobic region (13).

**Figure 3:**
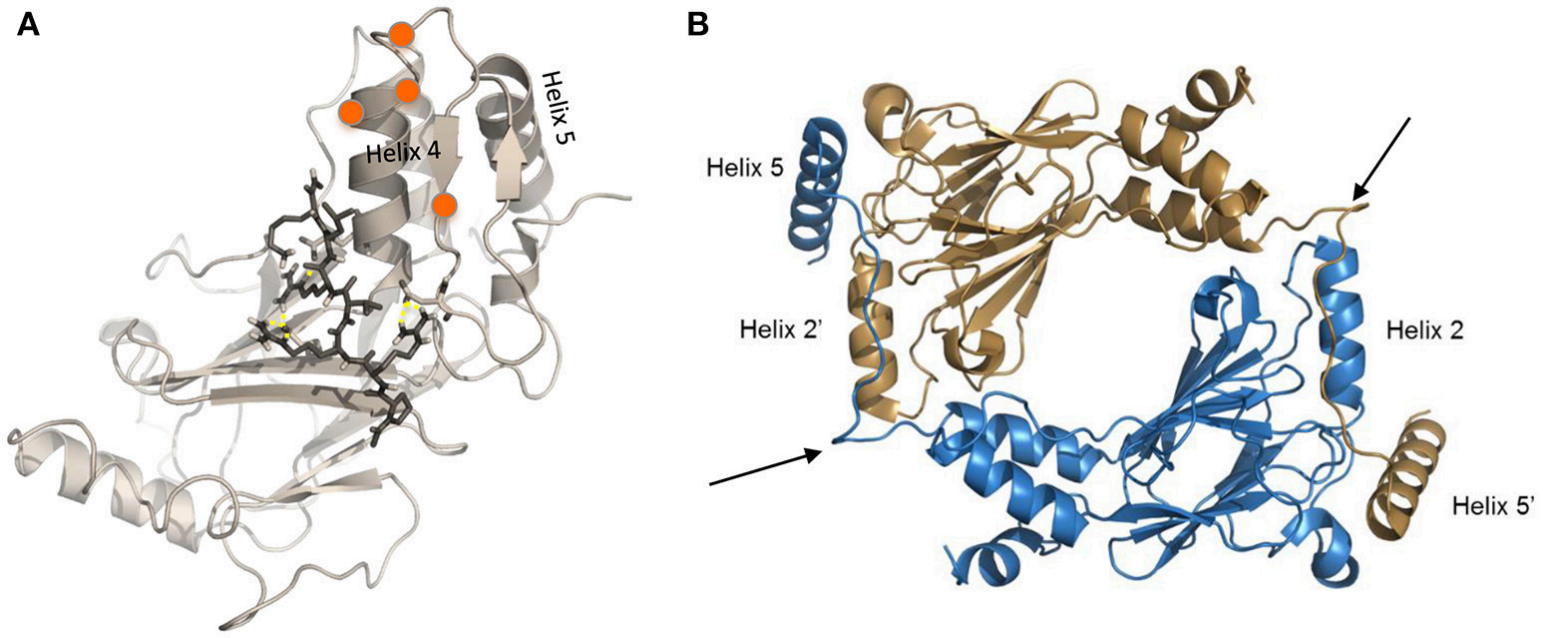
Modified crystal structures of IRF5. **(A)** Homology model of the inactive IRF5 C-terminal domain (variant 5) generated using the monomeric autoinhibited IRF3 C-terminal domain (PDB: 1QWT) as a template (113). Representative image from docking of an inhibitor (112) to the C-terminal SRR of the inactive IRF5 monomer, which results in maintenance of a closed, non-phosphorylated conformation. Orange balls represent phosphorylation sites at the C-terminal SRR. **(B)** Representative image generated from IRF5 crystal structure coordinates (13) showing formation of an IRF5 homodimer. Arrows show critical regions that are being therapeutically targeted to inhibit homodimerization between Helix 2 and Helix 5 (111).

Insights from the crystal structures, along with data from functional mutagenesis, provides key structural information that can be used to directly target each IRF family member. These models also allow for the further testing of different mechanisms that may lead to IRF activation and conformational changes that liberate the AID and expose critical residues essential for homo- or hetero-dimerization and other protein-protein interactions. Specific phosphorylated residues in the C-terminus, referred to as the serine rich region (SRR, Figure 2), contribute to the stabilization of IRF dimers and interaction with DNA. Mutational analysis of the SRR originally identified S425, S427, and S430 of the identical isoform encoded by *IRF5* variants 3 and 4 (Figure 2, red-bolded residues) as the critical sites of phosphorylation that are necessary for Newcastle disease virus (NDV)-induced IRF5 activation (101, 103). Later studies from multiple groups confirmed the functional importance of these three residues (13). While protein length and numerical amino acid location varies between IRF5 isoforms (104), the SRR is conserved (Figure 2). Given that we still do not know all of the pathways and mechanisms leading to IRF5 activation or inhibition of activation, further studies focused on identifying mutations that lead to either of these outcomes will be essential to our understanding of how better to target these molecules. An example of this was the finding years ago by others and us of dominant negative IRF mutants that lead to the inhibition of IRF transactivation function (104–110). These types of studies suggest that the utilization of small peptides that mimic the IRFs may lead to inhibition. Indeed, two examples of this currently exist for IRF5 that will be discussed in the last section [(111); U.S. Patent No US20160009772A1; (112); U.S. Patent No WO2017044855A2], but are depicted in Figure 3.

## IRF5 signaling pathways: the positive and negative paradigm

The combination of protein-protein interaction, signaling co-factors, adaptor proteins, and cell type specificity will all contribute to determining which IRF family member will be “turned on” in response to stimulation. For instance, IRF3 is ubiquitously expressed in all immune cells while IRF7 is more restricted in cells of lymphoid origin (101). IRF5, on the other hand, is expressed in monocytes, macrophages (Mφ), B cells and dendritic cells (DC) (16, 114).

**Figure 4:**
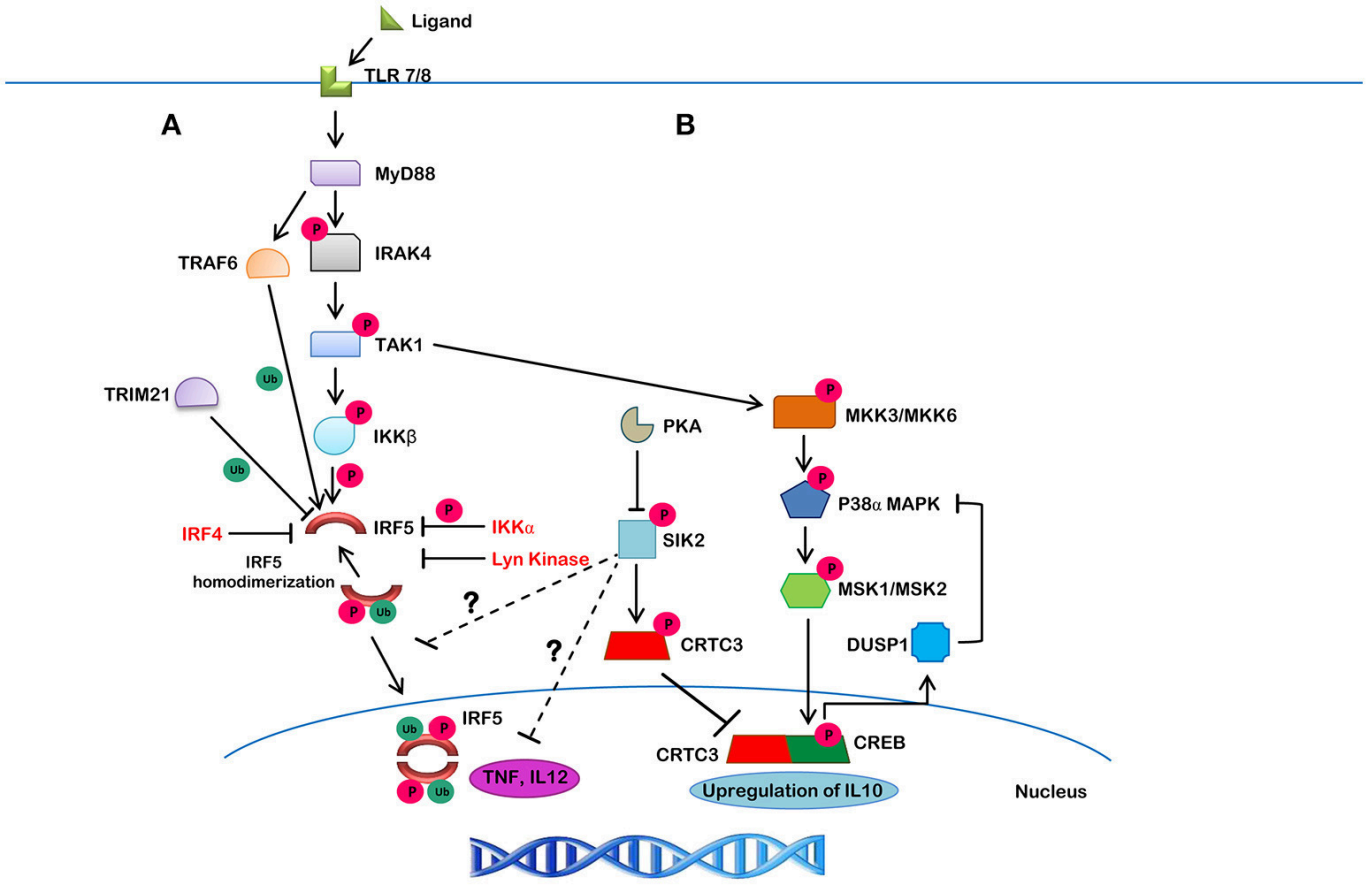
The canonical IRF5 signaling pathway and its negative regulation. **(A)** Upon ligand binding to TLR7/8, MyD88 gets recruited in, along with IRAK1/4 and TRAF6, which leads to the autophosphorylation of IRAK4 and ubiquitination of IRF5 by TRAF6. IRAK4 then activates TAK1, which then phosphorylates IKKβ. The ubiquitinated IRF5 is then phosphorylated by IKKβ (or other kinases). This action results in homodimerization and translocation of the IRF5 homodimer to the nucleus, leading to the production of downstream cytokines. Lyn kinase, IKKα and IRF4, on the other hand, were found to negatively regulate IRF5 activity. TRIM21 is a molecule that targets IRF5 for proteasomal- or lysosomal-mediated degradation. **(B)** A negative feedback loop may also be involved in the suppression of IRF5-mediated inflammatory gene transcription. TAK1 initiates a series of phosphorylation events on different kinases, including MMK3/MKK6, P38α/MAPK, MSK1/MSK2, and CREB, which leads to the upregulation of IL10. SIK2, on the other hand, inhibits CRTC3 activity by phosphorylation leading to its cytosolic localization and inhibition of IL10 expression. SIK2 also inhibits inflammatory molecules, such as TNF and IL12 by unknown mechanisms that may involve inhibition of IRF5 (shown by ?).

Innate pattern recognition receptors (PRRs), which include TLRs, C-type lectin receptors (CLRs), RIG-I-like receptors (RLRs), and NOD-like receptors (NLRs), all recognize various pathogen-associated molecular patterns (PAMPs) and danger-associated molecular patterns (DAMPs). In response to these PAMPs and DAMPs, intracellular signaling cascades are differentially triggered that induce the expression and/or activation of IRFs (115). In the case of TLR signaling, activation occurs via binding of ligand to receptor, leading to a conformational change that immediately recruits adaptor proteins. MyD88 is a proximal adaptor protein responsible for the propagation of the innate immune signal transduction downstream of TLR7 and upstream of IRF5 (9, 116). In the MyD88-dependent pathway, MyD88 recruits TNFR-associated factor 6 (TRAF6) and IL-1R-associated kinase 4 (IRAK4) followed by recruitment of IRAK1, IRAK2 or IRAK3 to form a complex called the Myddosome (117). IRF5 activation occurs downstream of this TLR7/8 pathway and has recently been shown to be phosphorylated by IKKβ (Figure 4), leading to downstream cytokine and chemokine expression (105, 118, 119). Additional reviews are available that cover in more detail the TLR-IRF signaling pathways (3, 11, 120, 121).

In human primary monocytes and macrophages, induction of IFNβ following infection of Staphylococcus aureus (RNA) was found to require two key signaling molecules in the TLR8-MyD88 pathway–TAK1 and IKKβ (122). Use of an IRAK4 inhibitor revealed that IRAK4 regulates TAK1 and IKKβ activity (123). Inhibition of IRAK4 autophosphorylation led to the inhibition of TAK1 activation, which resulted in the inhibition of IKKβ phosphorylation at S177, and inhibition of IRF5 activation and downstream proinflammatory cytokine production (123). IKKβ was previously identified as a kinase for IRF5 (Figure 2, blue-bolded serine) (118, 119).

As for negative regulators of IRF5 function, IRF4 was shown to act as an antagonist of IRF5 in Epstein-Barr Virus (EBV)-transformed cells (124). IRF4 knockdown resulted in elevated IRF5 expression. IRF4 was found to bind to similar IRF5 target genes and compete for binding with IRF5 (124). Further, a few studies reported that IRF4 also competes with IRF5 for MyD88 interaction, resulting in the negative regulation of downstream IRF5 targets (125, 126). While these are not direct effects on IRF5 itself, subsequent studies identified Lyn kinase as a direct regulator of IRF5 activity. Lyn kinase was found to bind to IRF5 and even phosphorylate it; however, phosphorylation did not alter protein activity (116). Instead, inhibition of IRF5 activity was due to the direct interaction of Lyn with IRF5 resulting in allosteric interaction. Further discussion of Lyn-IRF5 interaction is included below in the section on IRF5 post-translational modification.

Results from independent studies also allow us to speculate on other negative regulatory pathways of inflammatory cytokine expression that may regulate IRF5 (127, 128). For instance, SIK2 was reported to phosphorylate CRTC3, which results in its cytoplasmic localization and inhibition of IL10 expression. SIK2 has also been reported to downregulate TNF and IL12 production via an unknown mechanism. We speculate that components of this pathway may serve as a negative feedback loop that inhibits IRF5 activity (Figure 4). TRIM21-mediated dose-dependent degradation of IRF5 was also found to contribute to reduced IRF5 activity and may lead to a mechanism of inhibition (129).

## IRF5 post-translational modifications and key modifiers

Post-translational modifications (PTMs) are essential to protein stability and function. A single protein may undergo single or multiple reversible or irreversible PTM(s). Phosphorylation (of serine, threonine or tyrosine) is an important modification required by most IRFs for their activation and/or inhibition. IRFs also undergo either K48- (targeted for proteosomal degradation) or K63-ubiquitination (for intracellular trafficking). Here, we will discuss some of the most important modifiers and PTMs essential for IRF5 activation that could be potential targets for inhibition.

IRF5 can be phosphorylated by IKKβ which leads to homo-dimerization and nuclear translocation to induce IFN activation following viral infection (Figure 4) (118, 119). Phosphorylation is required not just for homo- and hetero-dimerization but also for the interaction with histone acetyltransferases (HATs) (70, 71, 130). Two independent studies identified IKKβ as a kinase that phosphorylates a single C-terminal Ser residue in IRF5 (Figure 2, blue-bolded serine) (118, 119). Mutation of this residue abrogated IRF5 homodimerization and nuclear translocation. Three additional Ser residues that were previously identified as being important for IRF5 activation (Figure 2, red-bolded serines) (101), may also be important for dimerization, based on crystal structure analysis (14, 15, 101, 103). These Ser residues, however, also appear to be essential to the liberation of the AID (13).

Prior to phosphorylation, IRF5 has been shown to undergo ubiquitination which is catalyzed by TRAF6 (98, 131). A few studies mentioned that ubiquitination is not required for IRF5 activation but it appears to be required for phosphorylation (116, 132). In particular, K410 and K411 are essential for IRF5 activation, nuclear translocation and the IFNα promoter-inducing activity (131). TRIM21 has been shown to ubiquitinate IRF5 which reduces or dose-dependently inhibits its activity via proteasomal- or lysosomal-mediated degradation (129).

Lyn kinase phosphorylates IRF5 at Y313 and Y335 but this modification was dispensable as transactivation ability of the double mutant IRF5 (YY313, 335FF) was still inhibited by Lyn (116). Further, a kinase-dead Lyn point mutant (K275D) inhibited IRF5 transcriptional activity. These data show that Lyn negatively regulates IRF5 transcriptional activity via a mechanism independent of its kinase activity and possibly via a direct interaction of Lyn with IRF5. IKKα also inhibits IRF5 function through phosphorylation which can be circumvented by the action of alkaline phosphatase causing it to undergo dephosphorylation (133).

Last, we previously reported that IRF5 activity may also be regulated by acetylation. We found that histone deacetylases (HDACs) and HATs CREB-binding protein (CBP)/p300 interact with IRF5 in response to virus infection, and this was required for IRF5 transactivation (15, 70, 130).

## Current therapeutic strategies to inhibit IRF5

IRF5 was identified as a key regulatory factor for macrophage polarization. The activation of IRF5 expression in macrophages decides their fate to either be M1 or M2 macrophages. Higher expression of IRF5 leads to M1 polarization whereas reduced or downregulated expression leads to M2 polarization (76). In a SCI mouse model, macrophage activation along with persistent inflammation was found to contribute to severity. After injury, there is an immediate influx of M2-activated macrophages; however, following this, there is a long-lasting phase characterized by an influx of activated M1 macrophages to the site of injury. This long-lasting phase of M1 macrophages causes derailed healing and compromises organ function(s) (92). Since up-regulated IRF5 expression induces the M1 macrophage phenotype, IRF5 siRNA was delivered *in vivo* by lipidoid nanoparticle to silence IRF5 in the macrophages that infiltrated the spinal cord injury wound. Nanoparticle-mediated IRF5 siRNA delivery to the wound resulted in a dramatic change in macrophage phenotype changing from M1 to M2 in the long-lasting phase. Decreased inflammation, attenuation of demyelination and neurofilament loss, and a significant improvement in locomotor function were found (92). A similar study using nanoparticle-mediated IRF5 siRNA delivery *in vivo* into macrophages residing in myocardial infarcts (MI) and in surgically induced skin wounds in mice showed resolution of inflammation and infarct healing. Furthermore, treatment led to the attenuation of post-MI heart failure after coronary ligation (81). Likewise, in the severe acute pancreatitis mouse model there is pancreatitis-induced activation of lung M1 macrophages with high expression of IRF5, TNFα, iNOS and IL10. These macrophages were polarized toward the M2 phenotype after treatment with IRF5 siRNA *in vitro*. Moreover, *in vivo*, treatment with IRF5 siRNA reversed the pancreatitis-induced activation of lung macrophages from M1 phenotype to M2 phenotype (134). Last, selective suppression of IRF5 in microglia cells using gene therapy with homing peptide-siRNA-IRF5 complexes in a mouse model of neuropathic pain resulted in a significant reduction in neuropathic pain (91).

An alternative method of targeting IRF5 was demonstrated using an AAG-rich microsatellite DNA mimicking oligodeoxynucleotide designated as MS19 to inhibit IRF5 activation. LPS stimulated RAW264.7 cells, when cultured along with MS19, resulted in reduced expression of iNOS, IL6, and TNFα along with inhibiting the nuclear translocation of IRF5 *in vitro* detected by western blot of nuclear and cytoplasmic extracts. Bioinformatics analysis revealed the mechanism of action of MS19 to be competition with IRF5 at regulatory consensus sequences in the promoter of target genes. MS19 was further studied in a murine model of septic peritonitis revealing that MS19 prolonged the survival of the mice and down-regulated the expression of iNOS, IRF5, IL6, and TNFα (135). Another interesting study using the natural polyphenol Mangiferin that is a component of Mangifera indica Linn. leaves found a marked reduction in IRF5 expression in macrophages stimulated with LPS/IFNγ. This translated into a significant reduction in pro-inflammatory cytokine expression (136). How Mangiferin down-regulates IRF5 expression is not currently known.

## New therapeutic strategies for targeting IRF5

Given its role in both innate and adaptive immune signaling, constitutive activation of IRF5, like other IRF family members, can create havoc on immune homeostasis leading to detrimental effects on cellular phenotypic plasticity and the development of autoimmune and inflammatory diseases. In this section, we discuss recent new methods that have been developed by our lab and others that directly target IRF5 activation and speculate on other possible avenues that may lead to IRF5 inhibition.

Some IRF family members regulate the expression and activity of other IRFs. Examples of this are seen with IRF1-IRF2 and IRF4-IRF5 (5, 104, 124, 137–139). These positive and negative feedback mechanisms show vulnerability in the signaling system that could be used for targeting. However, because of these mechanisms of co-regulation, altering the expression of individual IRF family members may lead to non-specific effects on other IRF family members. This may be cell type-dependent since not all IRFs are expressed in every cell type. An example of this methodology was used in cancer cells where inhibition of IRF2 expression/function was induced by upregulation of its antagonist, IRF1 (140). Similarly, IRF4 was identified as a negative regulator of IRF5 transactivation ability (106, 124, 126). Upregulation of these negative regulators would lead to a respective switch from pro-tumorigenic to anti-tumorigenic and pro-inflammatory to anti-inflammatory. Unfortunately, upregulation of these negative regulators may also impact other signaling pathways that could promote the development of other diseases depending on cell type. Another challenge to targeting the IRFs is targeting them in a cell type-specific manner.

Additional negative regulatory pathways of IRF5 are being discovered (127). SIK2 has been implicated as a negative regulator of TNF and IL12 production and CRTC3. Inhibition of CRTC3 prevents it from undergoing nuclear translocation and reduces IL10 expression. These data suggest that SIK2 plays a role in the regulation of pro- and anti-inflammatory signaling and may be a candidate to target therapeutically for the inhibition of autoimmune and inflammatory diseases. We are currently examining whether SIK2 may be a negative regulator of IRF5 (Figure 4).

IKKβ, IRAK1/4, and TRAF6 are activators of latent IRF5 that can also be targeted to inhibit its activity. Certainly, these have been the more common strategies in the Pharma industry since enzymes have catalytically active sites that are more readily accessible by small molecular weight compounds. Another possibility is the targeting of phosphatases that lead to the deactivation or inhibition of IRF5. These include A20 (132) and alkaline phosphatase (133). Again, similar to targeting kinases, phosphatases and ligases are not specific for one protein and therefore targeting them would be expected to lead to global changes in gene expression and protein activation. The same is true for other co-activators identified to interact with IRF5, such as CBP/p300 and GCN/PCAF; they are not specific to IRF5.

A number of viruses have now been shown to encode viral IRF (vIRF) homologs, including Kaposi's sarcoma-associated herpesvirus and rhesus monkey rhadinovirus, which function as dominant negative mutants by antagonizing IRF activity (110, 124, 141). Some of these dominant negative mutants lack the IRF DBD that do not allow them to bind to the host DNA, instead they form homo- and hetero-dimers with the IRFs leading to inhibition. Alternatively, C-terminal deletion mutants have been shown to inhibit IRF function by binding directly to host DNA, thus competing out wild-type IRFs (106–108). Although the mechanisms of dominant negative function have not been entirely worked out, given that most IRFs require homo- or hetero-dimerization for function, and/or interaction with other proteins, targeting these types of interactions would be expected to provide enhanced specificity. Additionally, other viral proteins have been found to inhibit IRF function through targeted degradation (142). These viral proteins, and/or sequences within them, may be further developed to inhibit IRF function.

In this regard, we and others have developed novel peptide inhibitors that utilize specific sequences within the IRF5 gene to inhibit activation. In collaboration with colleagues at Roche, a series of peptide inhibitors were developed based on crystal structure data predicting regions in the IRF5 protein that are critical for homo- and hetero-dimerization [(111); U.S. Patent No US20160009772A1]. We found that these inhibitors directly bind to the IRF5 protein, inhibit TLR-induced IRF5 homo-dimerization, nuclear translocation and downstream cytokine production. Independently, we developed another series of peptide inhibitors that are cell permeable, directly bind to full-length endogenous IRF5, and inhibit the development of murine lupus *in vivo* [(112); U.S. Patent No WO2017044855A2]. Results from these two studies support the specific targeting of IRF5 with inhibitors that directly bind to the protein. The value of targeting IRF5 directly rather than mediators of its activation is that specificity will be enhanced and inhibition will be independent of cell type and pathway of activation.

## Conclusions

Given the similarities in IRF crystal structures, mechanisms of activation and necessity of protein-protein interactions for activity, we expect that similar methodologies as those identified to inhibit IRF5 activation can be used to target other IRF family members.

## Author contributions

All authors listed have made a substantial, direct and intellectual contribution to the work, and approved it for publication.

### Conflict of interest statement

The authors declare they have a patent application related to IRF5 inhibitors (WO2017/044855A2).
